# Reduction in Floor Impact Noise Using Resilient Pads Composed of Machining Scraps

**DOI:** 10.3390/polym16202912

**Published:** 2024-10-16

**Authors:** Donghyeon Lee, Jonghoon Jeon, Wanseung Kim, Narae Kim, Minjung Lee, Junhong Park

**Affiliations:** 1 Department of Mechanical Engineering, Hanyang University, Seoul 04763, Republic of Korea; alajju3@hanyang.ac.kr (D.L.); rargon01@naver.com (J.J.); xqxwxexr@naver.com (W.K.); dogmecome@hanyang.ac.kr (N.K.); 2 Department of Architectural Engineering, Hanyang University, Seoul 04763, Republic of Korea; ajax3@naver.com

**Keywords:** floor impact noise, vibration reduction, resilient viscoelastic material

## Abstract

Floor impact noise is a significant social concern to secure a quiescent living space for multi-story building residents in South Korea. The floating floor, consisting of a concrete structure on resilient pads, is a specifically designed system to minimize noise transmission. This floating structure employs polymeric pads as the resilient materials. In this study, we investigated the utilization of helically shaped machining scraps as a resilient material for an alternative approach to floor noise reduction. The dynamic elastic modulus and loss factor of the scrap pads were measured using the vibration test method. The scrap pads exhibited a low dynamic elastic modulus and a high loss factor compared to the polymeric pads. Heavyweight impact sound experiments in an actual building were conducted to evaluate the noise reduction performance. The proposed pads showed excellent performance on the reduction in the structure-borne vibration of the concrete slab and resulting sound generation. The analytical model was used to simulate the response of the floating floor structure, enabling a parametric study to examine the effects of the resilient layer viscoelastic properties. Both experimental and analytical evidence confirmed that the proposed scrap pads contribute to the development of sustainable solutions for the minimization of floor impact noise.

## 1. Introduction

Apartments utilize concrete slab structures to separate each floor, thereby substantially enhancing spatial efficiency. While the structural design with concrete layers provides practical benefits, it is susceptible to impact noise transmission [1]. These noises adversely affect the quality of life for occupants on lower floors [2,3]. Consequently, the reduction in floor impact noise transmission is important to secure a quiescent residential space.

Heavyweight floor impact sound, a type of structure-borne noise, is characterized by a high intensity and prolonged duration [4]. To improve noise reduction performance, it is essential to reduce the structural vibration of the concrete slab, particularly in the low-frequency region [5]. This study focused on the application of floating floors, a specific implementation of constrained layer damping, to isolate the structural vibration. Several researchers have verified the damping effect of the floating floor and constrained layer. Sylwan [6] proposed an analytical model focusing on the compressional damping effect in sandwich structures with a viscoelastic core. Sisemore et al. [7] presented experiment results on the compressional damping in a cantilever sandwich beam. Kim et al. [8] utilized the Rayleigh–Ritz method to conduct a detailed analysis of the noise emission resulting from the vibration of sandwich panels. They proposed an analytical model, demonstrating that energy dissipation in the constrained layer is primarily due to compressional deformation. Schiavi [9] presented a constitutive model to calculate the improvement in impact sound insulation provided by floating floors. Branco et al. [10] conducted a comparative analysis of the acoustic performance achieved using various resilient materials such as lightweight mortar, expanded polystyrene, expanded cork, and clay granules. Caniato et al. [11] investigated the relationship between the viscoelastic properties of resilient materials and their acoustic performances. They conducted experimental studies to evaluate the noise reduction efficiency of the floating floor system.

To quantify the performance of the constrained layer, it is required to measure the viscoelastic properties of the resilient layer. Madigosky et al. [12] employed a wave propagation approach to measure the dynamic elastic modulus and the loss factor of the resilient materials. Subsequently, a method utilizing the longitudinal waves of a rod was proposed to measure the properties of polymers [13]. This method, which involves calculating the transmissibility function between two vibration responses of the specimen, was adopted as an ANSI standard [14]. Commonly used resilient materials for the interlayer include polymeric foams such as expanded polystyrene (EPS). However, the manufacturing process of these materials involves chemical additives that are gradually released into the air, and the foams undergo deformation under prolonged load. Their acoustical properties degrade over time, resulting in a decline in initial noise reduction performance [15,16]. Therefore, the suggestion of alternative materials becomes a crucial engineering problem to ensure a sustained vibration reduction performance throughout residential occupancy.

The requirement for eco-friendly vibration isolation materials has been a focus of research for decades. Rahman et al. [17] measured the properties of rubber-reinforced concrete beams. The dynamic modulus and vibration damping were examined using different mass configurations. Tiuc et al. [18] tested a mixture of recycled rubber particles and sawdust to evaluate its acoustic properties. The frequency of sound absorption and vibration damping performance varied depending on the type of specimen, regardless of the ratio and thickness of rubber and sawdust. Ribeiro et al. [19] proposed a floating floor structure using construction and demolition waste, underscoring the potential for sustainable approaches in the mitigation of floor impact noise.

Machining scraps are leftover materials generated during metal processing by machine tools. This waste material is typically recycled through melting processes. However, such processes incur substantial costs due to the high temperatures required to melt the scraps, leading to significant energy consumption. Additionally, the presence of contaminants such as cutting fluids necessitates further cleaning processes prior to recycling. These issues contribute to elevated energy expenses and can lead to atmospheric pollution [20]. Consequently, there has been research conducted into recycling methods for scraps [21]. In this study, we propose a sustainable approach to utilizing processing scrap in the construction of eco-friendly residential buildings. These scraps primarily contain iron or aluminum and come in various shapes depending on the machining conditions such as cutter type, processing direction, and the spindle rotation speed of the machine tool [22]. With sharp and unbreakable surfaces in a helical shape, machining scraps actively generate friction during vibration in contacted conditions. The vibration energy is dissipated through friction, which enhances vibration damping performance. Aslan et al. [23] investigated the mechanical properties of some recycled machining scraps by fabricating metal composites. The study applied different pressures to fabricate the samples, resulting in composites with higher hardness compared to bulk metal samples. Rongong et al. [24] measured the properties of metal swarf and applied them to vibration damping of a beam structure. Tang et al. [25] conducted experiments to measure the hysteresis of the tangled metal wire through periodic compression tests. The damping performance of the metal particles shows nonlinear behavior in reducing the vibration of box beams. These studies suggested the potential of machining scraps as vibration dampers. The utilization of the recyclable machining scraps in building structures provides solutions for both floor noise problems and the efficiency of material usage.

In this study, we proposed a resilient pad using recyclable machining scraps. The vibration test method was employed to measure the frequency-dependent behavior of the machining scrap pad. The results demonstrated that the proposed pad exhibited a higher damping performance than the polymeric pads. To further evaluate the practical performance, we constructed a floating floor structure incorporating the newly proposed machining scrap pads. Impact ball tests were conducted on the constructed floor, revealing improved damping performance across all frequencies compared to the existing resilient materials. A transverse compression analysis of the pad was employed to simulate the vibration damping of the resilient materials in a building structure. As the vibration amplitude increases, the nonlinearity in viscoelastic stress induces hysteresis in the dynamic behavior of the pad. The overall damping performance, incorporating both viscoelastic damping and hysteresis effects, was quantitatively evaluated using simulations based on the Duffing oscillator model. Both for the simulation and experiments, the damping performance of the proposed pad was validated in the frequency range of floor impact noise generation. The results highlight the potential of the machining scrap-based resilient material as an effective recycling application with enhanced vibration damping in building structures.

## 2. Properties and Performance Analysis of Recycled Metal Scrap Pads

### 2.1. Scrap Pads Made of Metal Recycled Materials

The spiral metal configuration imparts a distinctive shape to the clustered scrap, establishing favorable conditions for generating mutual friction in relation to each other during vibratory movement. This frictional interaction serves as a significant mechanism for impact energy dissipation. To utilize these scraps as the constrained layer between elastic structures, proper containment is required. The scraps were placed inside the box and enveloped in vinyl, forming a pad that maintained a constant size while allowing for relative movement during vibration.

**Figure 1:**
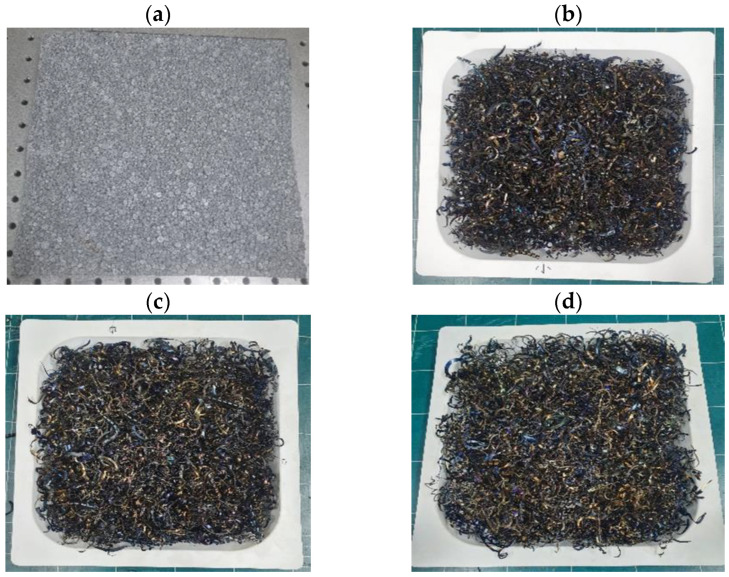
Image of the resilient materials including (**a**) conventional EPS pad and scrap pads with the same thickness of 3 mm but with different scrap lengths of (**b**) 60 mm, (**c**) 90 mm, and (**d**) 120 mm.

The viscoelastic properties of the scrap pads are influenced by the scrap geometric shape, which are determined by the workpiece and processing conditions. Figure 1 shows various resilient materials having different shapes. Our study compared the novel scrap pads with the widely used anti-vibration foam EPS pads. In particular, pads of different scrap lengths were used to understand the influence of the scrap length on the viscoelastic properties of these pads. The scrap specimens used in the experiments are composed of low-carbon steel and were wrapped in polyethylene vinyl, a packaging material, as shown in Figure 1.

### 2.2. Viscoelastic Properties of Scrap Pads

**Figure 2:**
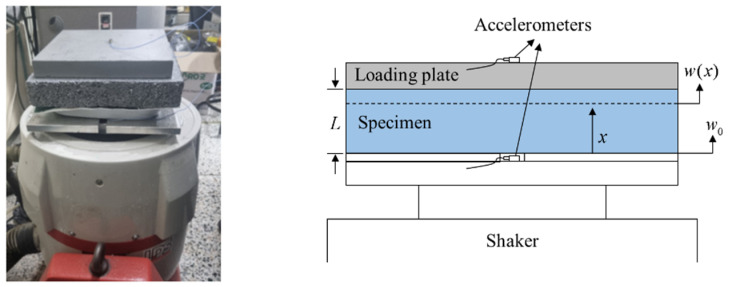
Experimental setup for measurement of the resilient material’s viscoelastic properties.

The vibration and noise reduction performance of the resilient material was determined by the viscoelastic properties—the frequency-dependent variation in the dynamic elastic modulus and loss factor. To obtain the viscoelastic properties of the metal scrap pads, the vibration experiment was conducted according to the ISO 9052-1 [26]. Figure 2 shows a schematic of the experimental setup for measuring the viscoelastic properties of the specimens. The entire system included the loading plate, specimen, shaker, and accelerometers. The weight of the loading steel plate was 8 kg with a cross-sectional area of 200 × 200 mm^2^. The area of the resilient material was equal to that of the loading plate. To transmit the random base excitation, an electric shaker (LDS V605, B&K, Virum, Denmark) was employed. Two accelerometers were used in the setup, one attached to the center of the base panel to measure the magnitude of the input vibration, and the other accelerometer was attached to the center of the steel plate to measure the transmitted vibration level. The base panel, specimen, and loading plate were attached by glue to prevent any separations during vibration.

The EPS pads and the scrap pads were tested in the experiments. Detailed information of the resilient pads is shown in Table 1. The natural frequency of the elastically supported system was determined during the process of optimizing the theoretical transmissibility function with the measured results [27], ${\hat{w}}_{x=L}/{\hat{w}}_{x=0}$. Utilizing the natural frequency, the dynamic stiffness s was obtained as [26]

(1) $$s=m{\left(2\pi f_{0}\right)}^{2}$$

where *f*_0_ is the natural frequency of the system and *m* is the mass of the loading plate. The loss factor was also obtained using the half-power bandwidth method [27].

(2) $$\eta =\left(f_{2}-f_{1}\right)/f_{0}$$

where *f*_1_ and *f*_2_ are the frequencies at which the magnitude of the transmissibility function attenuates by 3 dB below the peak value. From the dynamic stiffness, the dynamic elastic modulus of the resilient material was obtained using Hooke’s law [28].

(3) $$E=\left(sL\right)/A$$

where *A* is the cross-sectional area and *L* is the thickness of the resilient material. The acquired dynamic elastic modulus and loss factor enable an understanding of the damping performance for different specimens. Despite its widespread application, this method does not account for frequency-dependent variations. To investigate the frequency-dependent variation, an analysis of the longitudinal vibration of the specimens was conducted.

The equation of the one-dimensional vibration of the specimen is given as [29]

(4) $$\hat{E}{\hat{w}}_{xx}=\rho {\hat{w}}_{tt}$$

where *ŵ* is the displacement, *Ê* is the complex modulus, ρ is the density of the loading plate, and x is the coordinate from the base. Assuming harmonic vibration w^x,t=Rew^xeiwt, the vibration response is

(5) $$\hat{w}\left(x\right)=\hat{B}\cos\hat{k}x+\hat{C}\sin\hat{k}x$$

where $\hat{B}$ and Ĉ are the complex magnitudes and $\hat{k}=\rho w^{2}/\hat{E}$ is the complex wavenumber. To obtain the two unknown coefficients, two boundary conditions are imposed at *x* = 0 and *L* as

(6) $$\hat{w}\left(0\right)=w_{0},\;AE{\hat{w}}_{x}\left(L\right)=-m{\hat{w}}_{tt}\left(L\right)$$

From Equations (4) and (5), the transmissibility function at the points x=0 and *L* is given by

(7) $$\hat{w}\left(L\right)/\hat{w}\left(0\right)={\left(1-\cos\hat{k}L-\left(m/m_{r}\right)\hat{k}L\sin\hat{k}L\right)}^{-1}$$

where *m_r_* is the mass of the resilient material. The complex wavenumber $\hat{k}$ is obtained using the Newton–Raphson method. The complex modulus is calculated as

(8) $$\hat{E}=w^{2}\rho /{\hat{k}}^{2}=E\left(1+i\eta \right)$$

**Figure 3:**
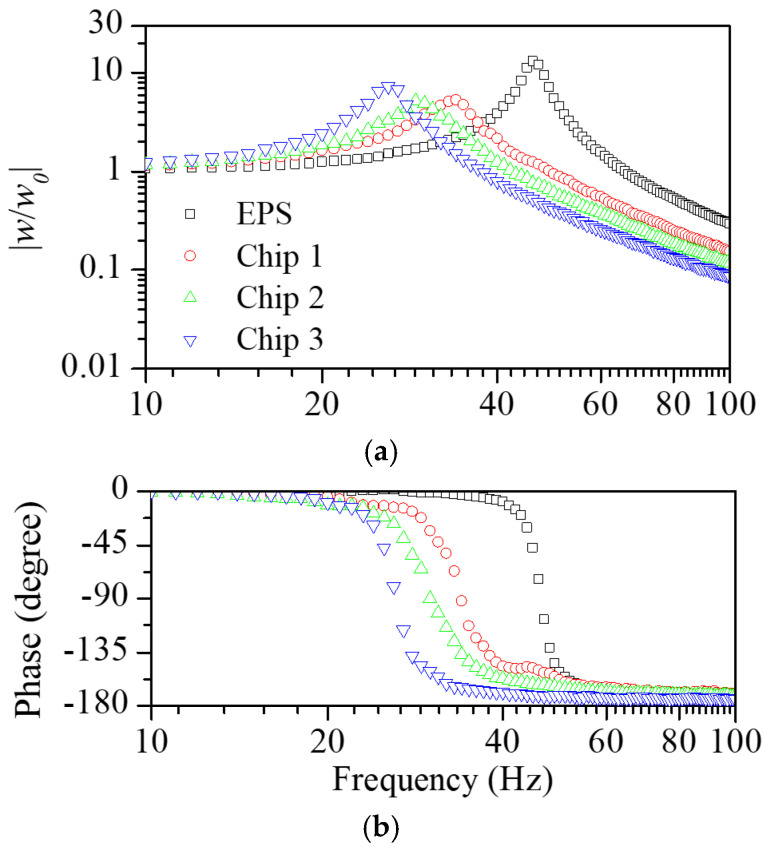
(**a**) Magnitude and (**b**) phase of the measured vibration transmissibility functions for the EPS and scraps in length variations.

**Figure 4:**
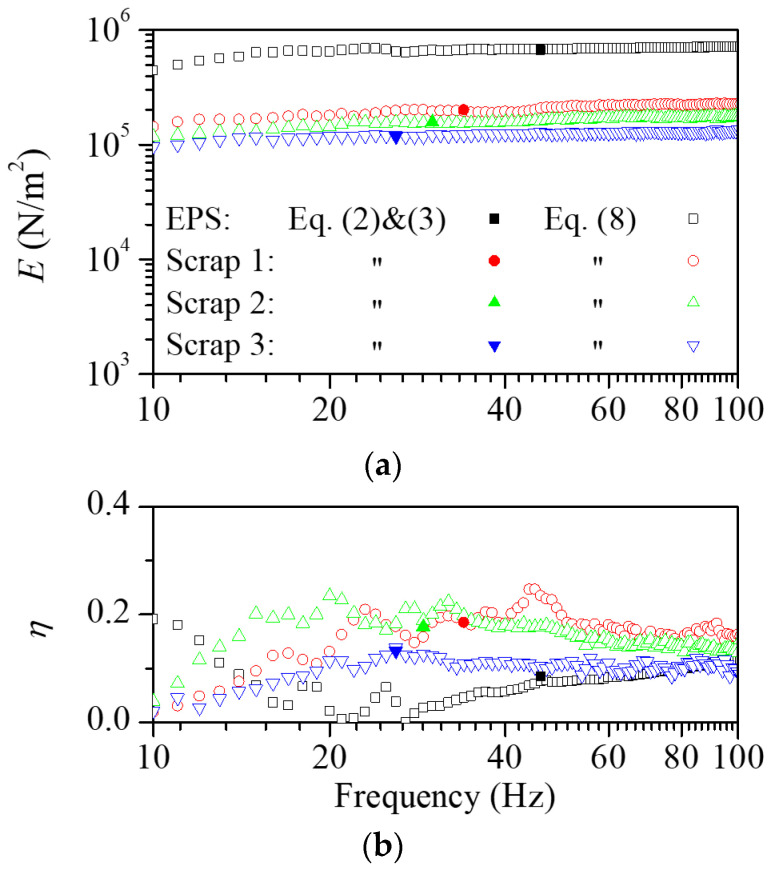
The estimated (**a**) dynamic elastic modulus and (**b**) loss factor for the EPS and scraps in length variations. The scrap pads showed a lower dynamic elastic modulus and a higher loss factor than the EPS pad. As the length of the scraps increased, the dynamic elastic modulus decreased while the loss factor increased.

Figure 3 shows the transmissibility function measured for the resilient materials considered in this study. In order to identify the difference in the properties depending on the scrap types, experiments were conducted using scrap pads in variations with scrap lengths of 60, 90, and 120 mm. Figure 4 shows the dynamic elastic modulus and loss factor obtained from the measured vibration transmissibility function. The dynamic elastic modulus of the machining scrap pads was lower than that of the EPS and decreased with the increasing length of the scraps. The loss factor of the scrap pads was greater than the EPS and exhibited a gradual decrease with the increasing scrap length.

**Figure 5:**
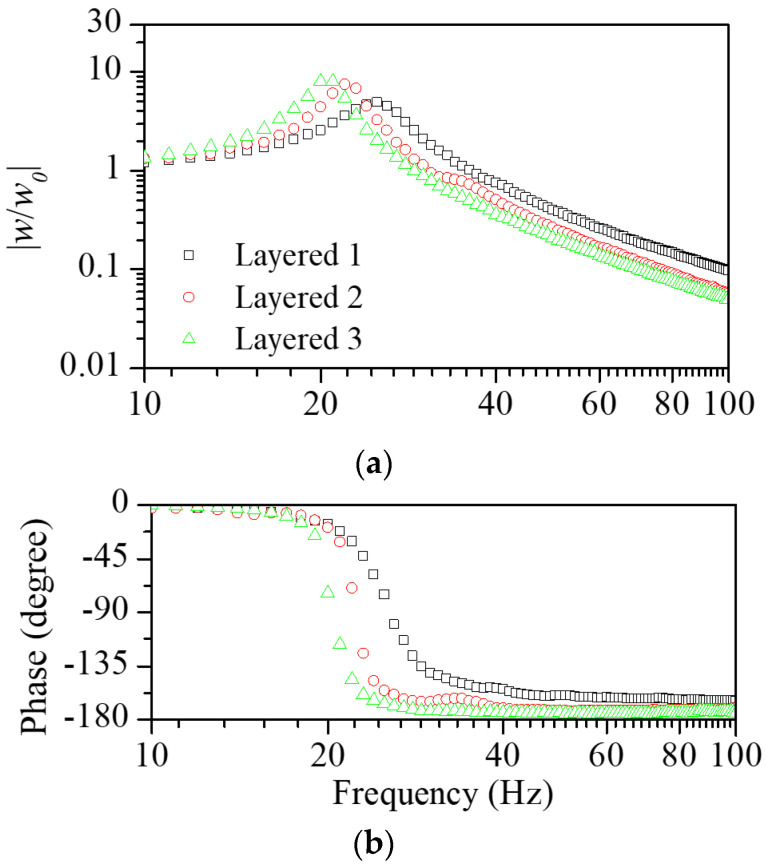
(**a**) Magnitude and (**b**) phase of measured vibration transmissibility functions for the EPS attached with scraps in length variations.

**Figure 6:**
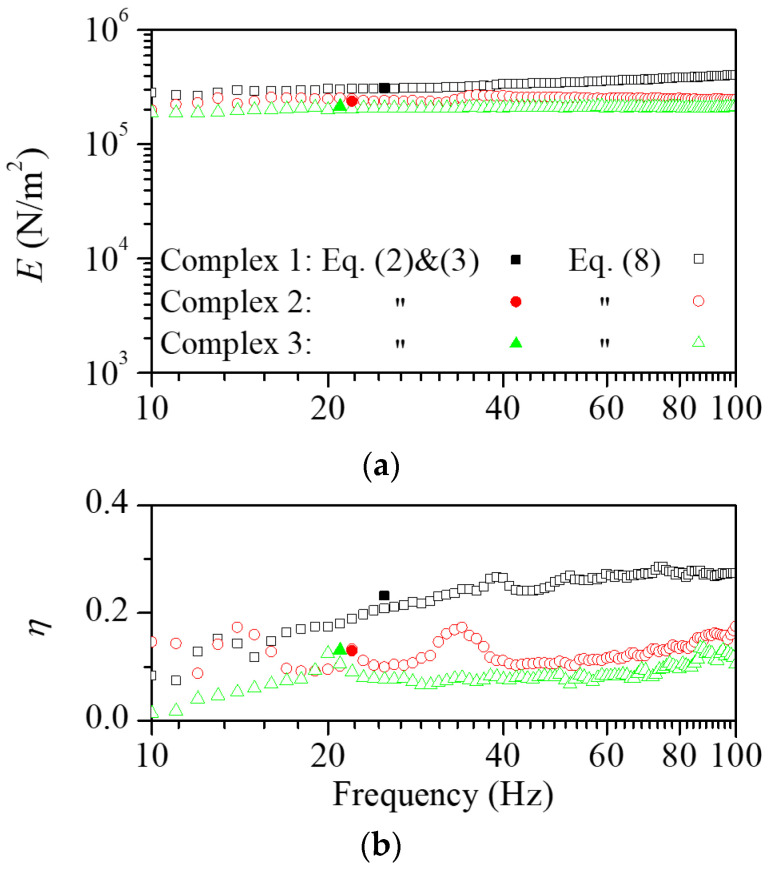
The estimated (**a**) dynamic elastic modulus and (**b**) loss factor for the EPS attached with scraps in length variations. Similar to the scrap pad cases, an increase in the length of the scraps resulted in a decrease in the dynamic elastic modulus and an increase in the loss factor.

For application in the floating structure, the layered scrap complex is required for easy and fast installations on the concrete slab. For this purpose, the layered scrap complex was built by mounting scrap pads on the EPS layer. Figure 5 shows the measured transmissibility function according to the scrap complex. The experiments were also conducted on the layered scrap complex with different scrap lengths of 60, 90, and 120 mm. Figure 6 shows the dynamic elastic modulus and loss factor calculated from the transmissibility function according to the layered scrap complex. Similar to the case of scrap pads, the dynamic elastic modulus and the loss factor decreased with the increasing length of the scraps. The elastic modulus was between the EPS pad and scrap pad. In all cases, the dynamic elastic modulus and the loss factor increased with increasing frequency due to the combined characteristics of EPS and scrap pads.

At the natural frequency, the dynamic elastic modulus exhibited notable similarity between the two estimation methods. The longitudinal vibration test method revealed frequency-dependent variation. For the EPS pad, the dynamic elastic modulus and the loss factor increased with increasing frequency. In the case of the scrap pads, the dynamic elastic modulus increased with frequency similar to typical polymeric materials, and the loss factor exhibited negligible frequency-dependent variation. These dynamic characteristics provide important information about vibration isolation performance for floor impact noise reduction.

**Figure 7:**
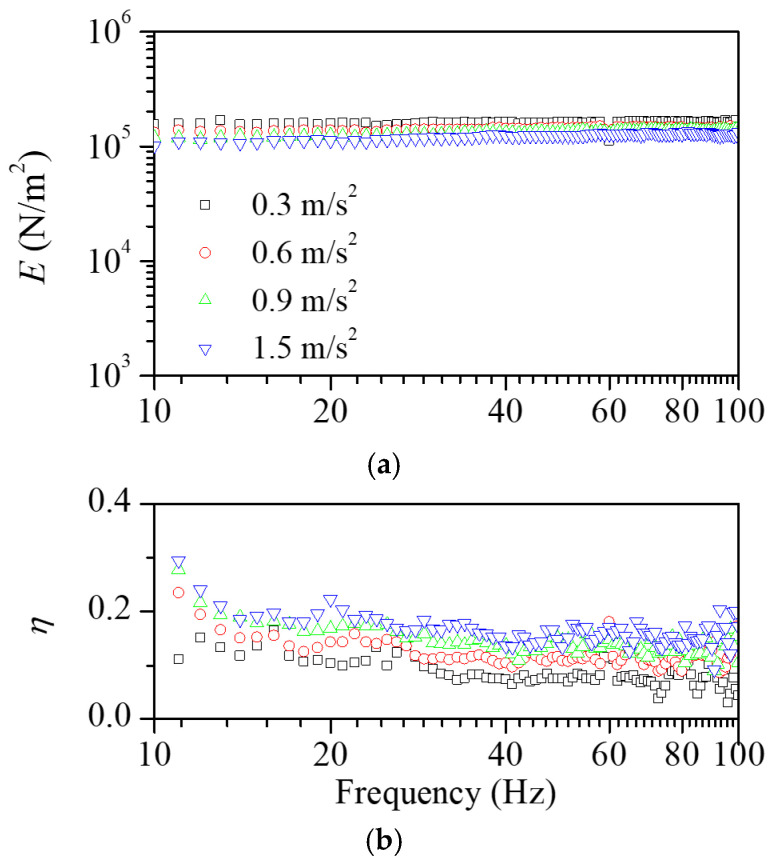
Dependence of (**a**) dynamic elastic modulus and (**b**) loss factor on the input vibration level for the scrap pad. As the excitation force increases, the dynamic elastic modulus decreases but the loss factor increases.

**Figure 8:**
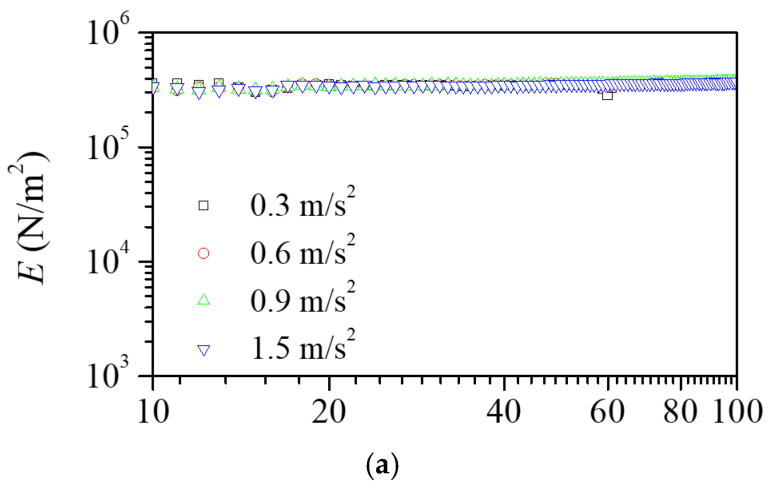

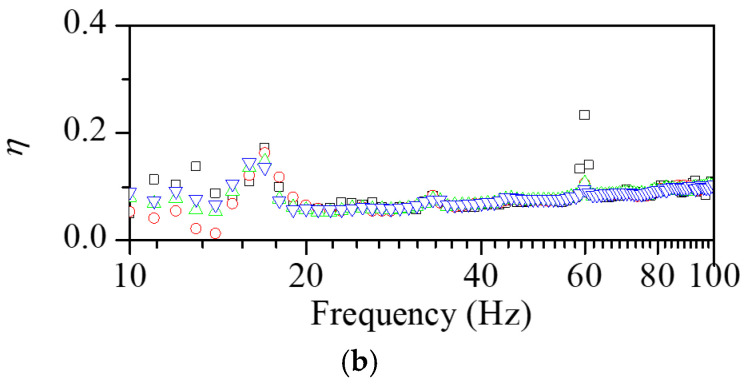
Dependence of (**a**) dynamic elastic modulus and (**b**) loss factor on the input vibration level for the EPS pad. There was no change in the viscoelastic properties with different excitation forces.

To investigate the nonlinear behavior of the machining scrap friction, experiments were conducted to find the effects of the vibration amplitudes. Figure 7 shows the influence of the vibration level on the dynamic elastic modulus and loss factor of the scrap pads. As the vibration level increased, the dynamic elastic modulus decreased, and the loss factor increased. Figure 8 shows the effects of the vibration level for the polymeric pads. There was no significant influence of the vibration amplitude on the viscoelastic properties. Overall, the result highlighted that great damping properties were achieved with a higher vibration level.

### 2.3. Quantification of Practical Damping Performance of Scrap Pads

In this study, a modified form of the Duffing oscillator equations was developed to better capture the system’s nonlinear dynamics. The nonlinear restoring force, dependent on elasticity varying with the vibration level, replaced the conventional cubic (third-order) nonlinear term in the equations. The normalized form of the equations is given by

(9) $$\ddot{x}+\left(L/A\right)\left\{E^{*}\left(x\right)\left(1+i\eta \right)\right\}x=F/m$$

where *x* represents the displacement and E* denotes the normalized dynamic modulus, which is dependent on the vibration level. This modification provides a more accurate representation of the system’s softening behavior under increasing excitation.

**Figure 9:**
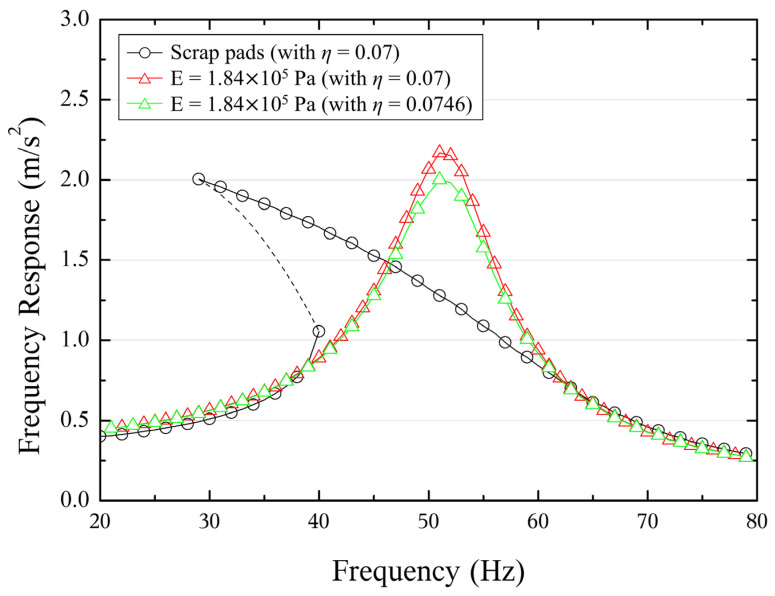
Frequency response of Duffing oscillator problem with the nonlinear dynamic modulus of the scrap pad and equivalent modulus with a constant loss factor, analyzed at a vibration level of 1.5 m/s^2^.

Using the modified Duffing equation, the frequency response functions of both the linear stiffness system and the nonlinear stiffness system with the same loss factor were numerically analyzed. The differences in the levels of these frequency response functions were interpreted as additional damping effects. Figure 9 presents the frequency responses of a single-degree-of-freedom system with both the nonlinear dynamic modulus and the equivalent linear modulus (*E_eq_* = 183,700 Pa). In the system with the nonlinear modulus, excited at a vibration level of 1.5 m/s², the response magnitude can be matched by substituting a higher damping factor (η* = 0.0746) into the equivalent linear modulus system, which originally had *η* = 0.07. Notably, as observed in Table 2, the additional damping effect diminishes as the vibration level increases. Due to the reduction in the dynamic modulus, the resonance range shifts to lower frequencies, leading to larger displacements that further amplify the system’s softening behavior.

## 3. Floor Impact Noise Reduction by Scrap Pads

### 3.1. Experimental Setup to Measure Floor Impact Noise in Building Structures

**Figure 10:**
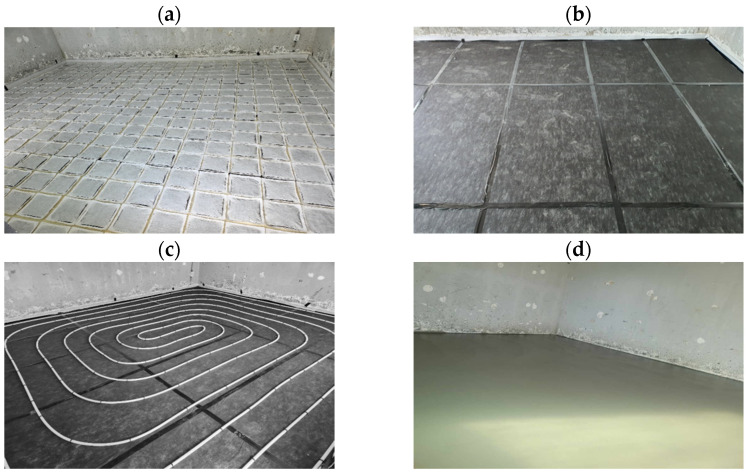
Installation of the floating floor incorporating the layered scrap complex: (**a**) scrap pads, (**b**) EPS pads, (**c**) heating plumbing, and (**d**) finishing mortar.

**Figure 11:**
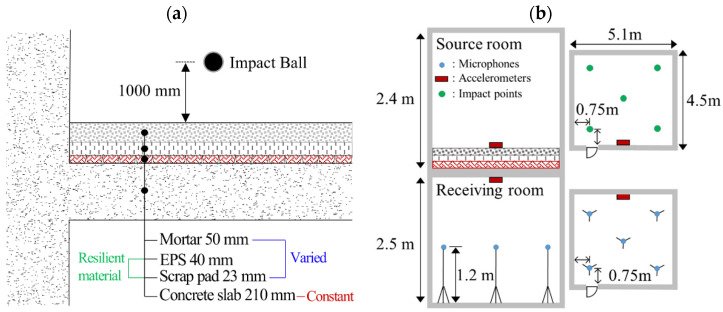
(**a**) Floor cross-sectrion and (**b**) microphone location schematic of the experimental setup for the floor impact noise measurement of the transmitted sound level. The microphones were placed at a distance of 0.75 m from the center and four corners. The source room and receiving room have an area of 22.95 m^2^ ( 5.1 × 4.5 m^2^) with heights of 2.5 m and 2.4 m, respectively.

In order to assess the performance of the floating floor structure with the interlayer including the fabricated machining scrap pads, a heavyweight impact experiment was conducted in an actual building structure. Figure 10 shows the installation process of the floating floor. The foundational concrete slab was 210 mm in thickness with a density of 2300 kg/m^3^. A 63 mm thick interlayer consisting of EPS and scrap pads was arranged on the slab. On this interlayer, a 50 mm thick finishing mortar with a density of 2000 kg/m^3^ was mounted as the floor structure. The machining scrap and EPS pads were fixed in place to ensure their positional stability. The configuration of the floating floor structure with machining scrap and EPS pads is shown in Figure 11a.

For the heavyweight impact test, we conducted impact excitation using a ball being dropped from a height of one meter on the source room. The material, shape, mass, and coefficient of restitution of the ball are designed according to the KS F ISO 10140-5 [30]. To measure the vibration magnitude of the source room and the vibration transmitted to the receiving room, the accelerometers (Dytran, 3056D1, Virum, Denmark) were attached to the center of the ceiling of the receiving room and the center of the source room, as shown in Figure 11b. In the receiving room, five microphones (B&K, Type 4191) were installed to measure the sound.

### 3.2. Floor Impact Sound Insulation Performance

**Figure 12:**
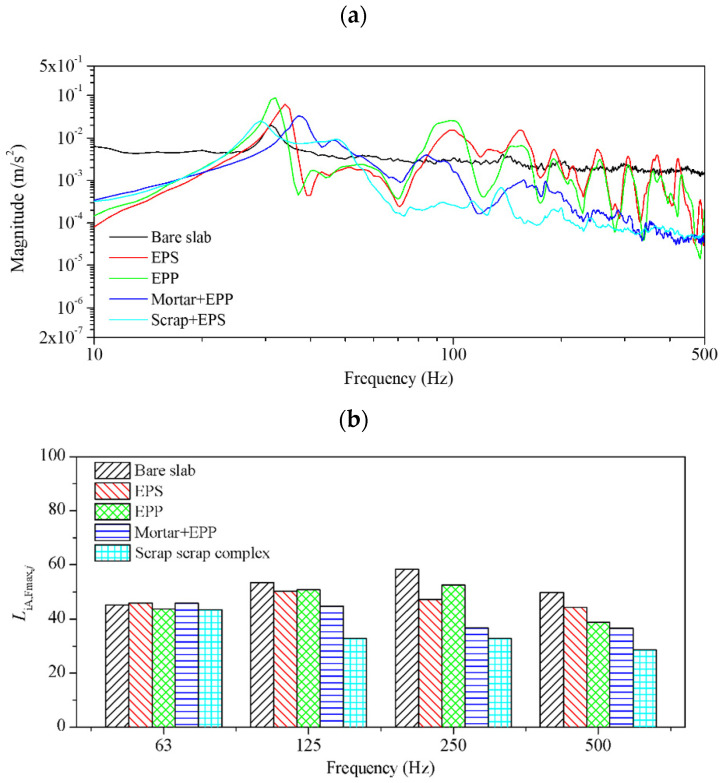
Heavyweight impact sound insulation performance: (**a**) vibration magnitude and (**b**) A-weighted sound pressure level in octave bands.

The vibration magnitude and A-weighted maximum floor impact sound level ($L_{\mathrm{iA},\mathrm{Fmax}}$) of the EPS and scrap layer were evaluated in comparison with different interlayers. Layered scrap complex 1, which exhibited the highest loss factor in the vibration test, was selected as the test specimen. It was compared with EPS and EPP, which are widely used as vibration isolation materials in construction. The frequency response of vibrations transmitted to the receiving room is shown in Figure 12a. For the structural vibration transmission, the structures constructed with the layered scrap complex exhibited a low resonance frequency and improved structural vibration reduction performance on the entire frequency ranges compared to structures mounted with EPS and EPP. The A-weighted maximum floor impact sound level, a single number quantity used to determine floor impact noise performance, was determined by utilizing the heavyweight impact sound pressure level within octave bands. This calculation was conducted in accordance with the standards ISO 10140-3 and ISO 717-2 [31,32], as outlined by the following equation:

(10) $$L_{\mathrm{iA},\mathrm{Fmax}}\;=10\log\sum 10^{\left(L_{i,F\max,j},+,A_{j}\right)/10}$$

where $L_{i,Fmax,j}$ is the sound pressure level averaged for all excitation positions and measurement points, *A_j_* is the A-weighted correction value corresponding to the 1/1 octave band, and *j* represents each 1/1 octave band in the range of 50 Hz to 630 Hz. The measured sound pressure level in octave bands is shown in Figure 12b. Similar to the vibration responses, noise reduction performance was verified across all frequency bands. The A-weighted maximum floor impact sound level was 60.2 dB for bare slabs, 47.3 dB for the EPS pads, and 44.2 dB for the scrap complex. The scrap complex showed the best performance in vibration and noise reduction in the actual building. The structure of the resilient layer and the calculated A-weighted maximum floor impact sound levels are shown in Table 3.

## 4. Prediction of Floor Impact Noise Reduction Performance

**Figure 13:**
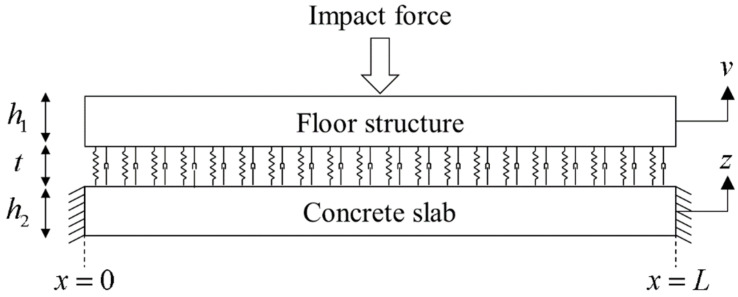
Elastic–viscoelastic–elastic sandwich structure with compressional damping for the floating floor structure.

The floating floor structure with vibration-resilient material was represented by the sandwich structure by analyzing the compressional damping model, as shown in Figure 13. The equations of motion of the elastic layers are represented as follows [33]:

(11a) $$-E_{1}I_{1}v_{xxxx}=k^{*}\left(v-z\right)+\rho_{1}bh_{1}v_{tt}$$

(11b) $$-E_{2}I_{2}z_{xxxx}=k^{*}\left(z-v\right)+\rho_{2}bh_{2}z_{tt}$$

where *E*_1_ and *E*_2_ are the elastic modulus, *I*_1_ and *I*_2_ are the moments of the inertia, and $\rho_{1}$ and $\rho_{2}$ are the densities of the floor structure and concrete slab. k* is the complex dynamic stiffness per unit length of the resilient viscoelastic layer.

Assuming a harmonic vibration, the solution was expressed as

(12a) $$v\left(x\right)=\overset{2}{\underset{k=1}{\sum }}A_{1k}\sin\varepsilon_{k}x+A_{2k}\cos\varepsilon_{k}x+A_{3k}e^{\varepsilon_{k}\left(x-L\right)}+A_{4k}e^{-\varepsilon_{k}x}$$

(12b) $$z\left(x\right)=\sum_{k=1}^{2}M_{k}\left(A_{1k}\sin\varepsilon_{k}x+A_{2k}\cos\varepsilon_{k}x+A_{3k}e^{\varepsilon_{k}\left(x-L\right)}+A_{4k}e^{-\varepsilon_{k}x}\right)$$

where

(13a) $$\varepsilon_{1,2}={\left[-\alpha /2\mp \sqrt{\alpha^{2}/4-\eta }\right]}^{\frac{1}{4}}$$

(13b) $$\alpha =\left(k^{*}-\rho_{1}\omega^{2}\right)/E_{1}I_{1}+\left(k^{*}-\rho_{2}\omega^{2}\right)/E_{2}I_{2}$$

(13c) $$\eta =\left[\rho_{1}\rho_{2}\omega^{4}-k^{*}\omega^{2}\left(\rho_{1}+\rho_{2}\right)\right]/E_{1}I_{1}E_{2}I_{2}$$

(13d) $$M_{1}=\left(k^{*}-\rho_{1}\omega^{2}+E_{1}I_{1}{\varepsilon_{1}}^{4}\right)/k^{*}$$

(13e) $$M_{2}=\left(k^{*}-\rho_{1}\omega^{2}+E_{1}I_{1}{\varepsilon_{2}}^{4}\right)/k^{*}$$

For the floor structure, we applied free boundary conditions on the floating floor and fixed boundary conditions on the concrete slab. The impact force was applied to the center of the floor structure. The constraint matrix and loading matrix were obtained, and the coefficients were determined through an inverse matrix computation.

The vibration analysis of the floating floor structure was performed. The results showed the performance of the impact noise reduction by the resilient interlayer. The mechanical properties of the structure were $\rho_{1}$ = 2000 kg/m^3^, *E*_1_ = 1.63 × 10^10^ N/m^2^, *h*_1_ = 50 mm, $\rho_{2}=2300\;\mathrm{kg}/m^{3}$, *E*_2_ = 2.48 × 10^10^ N/m^2^, *h*_2_ = 210 mm, *t* = 50 mm, and *L* = 4.5 m. The measured dynamic properties of layered scrap complex 1 from the vibration tests were used to investigate the effects of the resilient layer compressional stiffness, k*.

**Figure 14:**
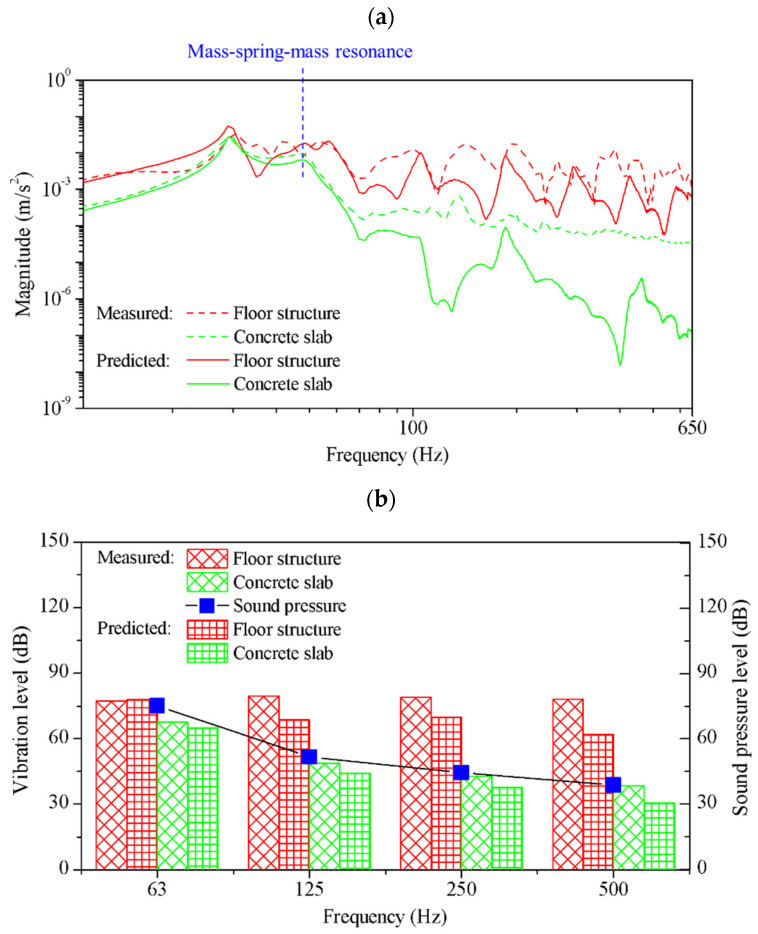
Comparison between the measured and predicted vibration responses of the floating floor structure to identify the influence of the resilient layer: (**a**) vibration response in narrow bands and (**b**) levels in octave bands.

**Figure 15:**
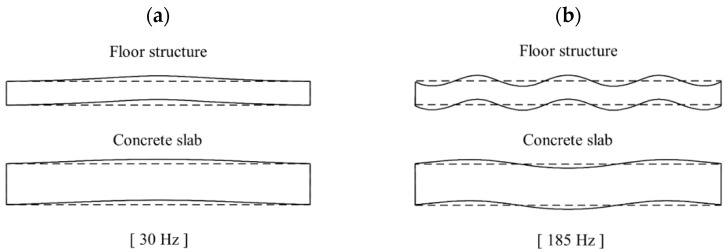
The vibration mode shape of the floating floor structure: (**a**) in-phase mode observed at 32 Hz and (**b**) out-of-phase mode observed at 185 Hz.

Figure 14a shows the measured and predicted vibration response of the floating floor structure. To evaluate the effectiveness in reducing the vibration generation, the vibration response of the floor structure and the concrete slab were compared. The predicted response was similar to the measured values obtained in the actual building experiments, although two-dimensional wave propagations were neglected in the simulation. The first natural frequency was identified as 30 Hz. Within this frequency range, the concrete slab vibrated in-phase to those of the floor structure. The mode shape observed at 30 Hz of the floor structure and concrete slab is shown in Figure 15a. Due to the same magnitude vibrations in this frequency range, negligible damping occurred in the resilient layer. The compressional interaction between the elastic layers and the viscoelastic layer occurred at 47 Hz. The frequency was calculated by the following equation for mass–spring–mass system resonance:

(14) $$f_{comp}=\sqrt{k^{*}\rho_{1}h_{1}\rho_{2}h_{2}/\left(\rho_{1}h_{1}+\rho_{2}h_{2}\right)}/2\pi =45.8\mathrm{Hz}$$

In this frequency, the vibration of the concrete slab was amplified due to resonance compared to those without the floor structure. Above this mass–spring–mass resonance frequency, the two elastic layers vibrated out-of-phase. These incoherent vibrations led to deformation of the resilient layer (scrap pads), which induced the compressional damping effect. The mode shape observed at 185 Hz of the floor structure and concrete slab is shown in Figure 15b.

Figure 14b shows the comparison between measured and predicted vibration levels in octave bands. Both measured and predicted results confirmed a rapid decline in vibration levels from the center frequency of 125 Hz, which was larger than the resonance frequency of the mass–spring–mass system. The figure also shows a correlation between the slab vibration levels and the measured sound pressure levels in octave bands. With an increase in frequency, the attenuation rates of the sound pressure level were found to be commensurate with the vibration level. This consistency reinforces the mechanism that reducing structural vibrations in concrete slabs through compressional damping contributes to minimizing heavyweight impact sound generation.

**Figure 16:**
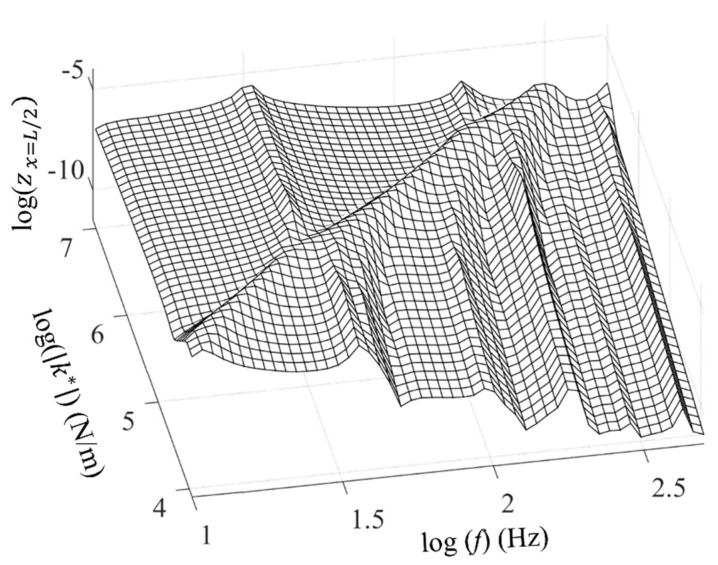
Effects of complex dynamic stiffness on frequency response of the concrete slab displacement.

**Figure 17:**
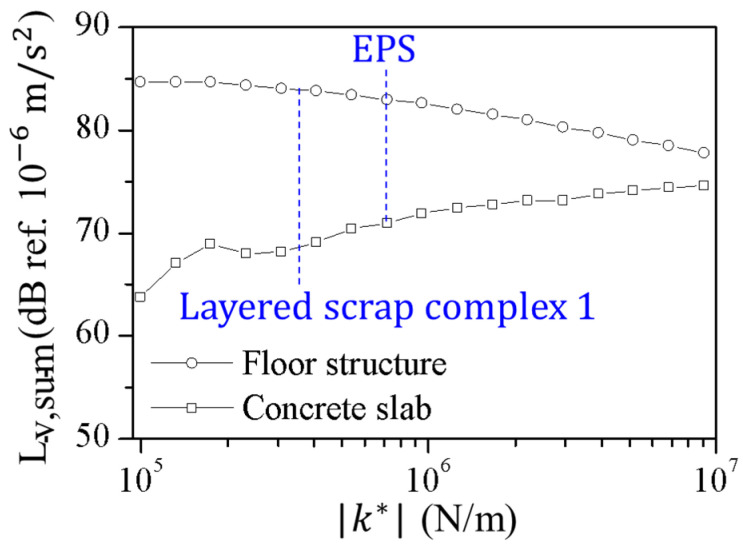
The predicted vibration level from the concrete slab as a function of the elastic dynamic stiffness of the resilient layer for the floor structure and concrete slab.

Figure 16 shows the influence of compressional stiffness on the vibration response of a concrete slab. The mass–spring–mass resonance frequency was proportional to the stiffness of the resilient layer. Above the mass–spring–mass resonance frequency, the incoherent vibrations of the elastic layers induced compressional damping. Figure 17 shows the predicted vibration level according to the elastic dynamic stiffness of the resilient material. A low dynamic elastic stiffness results in reduced vibration levels in the concrete slab and an increased vibration level in the floor structure. The low dynamic elastic stiffness decreased the mass–spring–mass resonance frequency and broadened the frequency range where the compressional damping effect was pronounced. However, excessively low stiffness increased the vibration level of the floor structure and led to structural instability. The scrap pad introduced in this study, characterized by its low stiffness and high loss factor compared to conventional polymer foam pads, demonstrated superior vibration damping capabilities, particularly when subjected to impulsive and large-amplitude vibrational forces. Additionally, the incorporation of steel in the pad’s composition provided static rigidity at the occurrence of high displacement, ensuring structural stability.

## 5. Conclusions

In this study, the use of the recycled machining scrap pad constructed as a resilient material to efficiently reduce heavyweight impact noise and vibration was presented. The scrap pad was placed as the resilient interlayer of a floating floor structure for actual installation in building structures. During the vibration transmission, the transverse movements induced compressional deformation of the resilient layer. Low stiffness with high damping was required for the efficient isolation of impulsive vibration responses and noise generation. Through the proposed vibration test method, the viscoelastic properties of the scrap pads were measured. The scrap pads exhibited a low dynamic elastic modulus with a high loss factor compared to polymeric foam pads. Notably, this effect becomes pronounced with a larger-amplitude vibration. To access the practical effectiveness in actual buildings, we implemented the fabricated layered scrap complex in the building floor structure. The impact ball test was employed to measure the transmitted structural vibrations and noise across floors. The performance of machining scrap pads was evaluated against polymeric pads by comparing the A-weighted maximum floor impact sound level. The measured results indicate that the use of machining scrap significantly outperforms existing polymeric resilient materials in reducing floor vibration and noise. To investigate the mechanism of noise reduction, a vibration simulation was conducted using the numerical model of the floor structures with the compressional resilient layer. With the viscoelastic properties obtained from the proposed vibration test measurements, it was confirmed that compressional deformation and the resulting damping influence the noise reduction performance. The effect of the viscoelastic properties on the vibration transmission was detailed via parametric studies. This analysis highlighted the potential of recycled machining scrap pads as an alternative for vibration and noise reduction in building structures.

## Figures and Tables

**Table 1:** Parameters of the resilient pads used for the viscoelastic property measurement experiments.

Resilient Pad	Scrap Length (mm)	Pad Thickness (mm)	Mass (g/m^2^)
EPS	-	40	875
Scrap 1	60	23	4550
Scrap 2	90	23	4600
Scrap 3	120	23	4650
Layered scrap complex 1	60	63 (Scrap 1 + EPS)	5425
Layered scrap complex 2	90	63 (Scrap 2 + EPS)	5475
Layered scrap complex 3	120	63 (Scrap 3 + EPS)	5525

**Table 2:** Numerically derived practical loss factor corresponding to the excited vibration level.

Vibration Level (m/s^2^/kg)	Practical Loss Factor, η*
0.3	0.0807
0.6	0.0812
0.9	0.0824
1.2	0.0812
1.5	0.0746

**Table 3:** Layer structure and A-weighted maximum floor impact sound level.

Resilient Pad	Mortar Thickness (mm)	Total Thickness (mm)	$L_{iA,Fmax}$ (dBA)
- (Bare slab)	-		60.2
EPS (40 mm)	70	110	53.5
EPP (40 mm)	70	110	55.2
Mortar (30 mm) + EPP (40 mm)	50	120	48.9
Scrap complex: Scrap (23 mm) + EPS (40 mm)	50	113	44.2

## Data Availability

The original contributions presented in the study are included in the article; further inquiries can be directed to the corresponding author.
